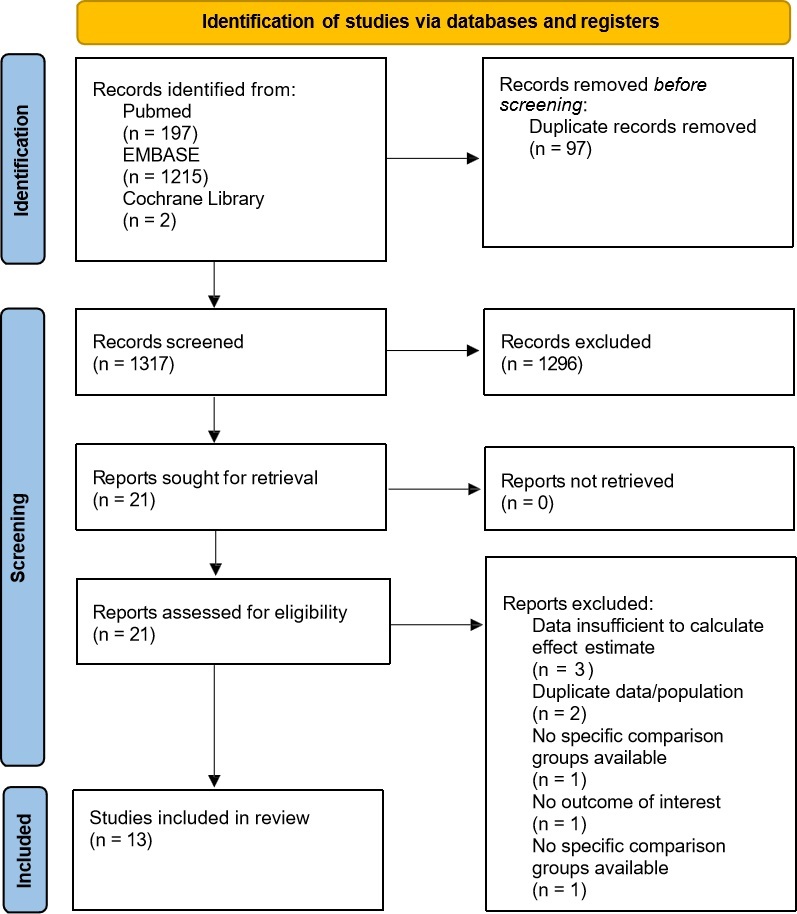# Effect of pretransplant dialysis modalities on pancreas-kidney transplant outcomes: a systematic review and meta-analysis

**DOI:** 10.1097/JS9.0000000000001542

**Published:** 2024-05-03

**Authors:** Yue Li, Yangming Tang, Yu Fan, Tao Lin, Turun Song

**Affiliations:** a Department of Urology, Institute of Urology, West China Hospital, Sichuan University; b Transplant Center, West China Hospital, Sichuan University, Chengdu, People’s Republic of China

**Keywords:** hemodialysis, pancreas-kidney transplantation, peritoneal dialysis, prognosis, systematic review

## Abstract

**Background:**

The impact of different pretransplant dialysis modalities on post-transplant outcomes for pancreas-kidney transplantation is currently unclear. This study aims to assess the association between pretransplant dialysis modalities [hemodialysis (HD) and peritoneal dialysis] and outcomes following pancreas-kidney transplantation.

**Methods:**

The authors searched PubMed, EMBASE, and the Cochrane Library for relevant studies published from inception until 1 December 2023. The authors included studies that examined the relationship between pretransplant dialysis modalities and clinical outcomes for pancreas-kidney transplantation. The primary outcomes considered were patient, pancreas and kidney graft survival, and intra-abdominal infection.

**Results:**

A total of 13 studies involving 1503 pancreas-kidney transplant recipients were included. Pretransplant HD was associated with improved pancreas graft survival (hazard ratio = 0.71, 95% confidence interval: 0.51–0.99, *I*²=12%) and a decreased risk of intra-abdominal infection [odds ratio (OR)=0.69, 95% CI: 0.51–0.93, *I*²=5%). However, no significant association was found between the dialysis modalities and patient or kidney graft survival. Furthermore, pretransplant HD was linked to a reduced risk of anastomotic leak (OR=0.32, 95% CI: 0.161–0.68, *I*²=0%) and graft thrombosis (OR=0.56, 95% CI: 0.33–0.96, *I*²=20%).

**Conclusion:**

Pretransplant HD is the preferred dialysis modality while awaiting pancreas-kidney transplantation, although well-designed prospective studies are needed to confirm these findings.

## Introduction

Highlights

- The effect of pretransplant dialysis modalities on post-transplant outcomes in patients receiving pancreas-kidney transplantation is uncertain.
- In this systematic review and meta-analysis of 13 observational studies involving 1503 patients, patients undergoing hemodialysis had a significantly decreased risk of pancreas graft failure, intra-abdominal infection, anastomotic leak, and graft thrombosis than patients undergoing peritoneal dialysis.
- The findings suggest that hemodialysis is the preferred dialysis mode of choice during the waiting period for pancreas-kidney transplantation.

Simultaneous pancreas-kidney (SPK) or pancreas after kidney (PAK) transplantation is the preferred treatment for patients with insulin-dependent diabetes mellitus and end-stage renal disease, because it restores long-term glycemic control and can reduce secondary diabetic complications^1–3^. Compared to insulin replacement, pancreas transplantation currently remains the most effective and reliable way to achieve sustained normal blood glucose, prevent hypoglycemia and ketoacidosis, and normalize hemoglobin A1c levels^4^. However, the number of pancreas transplants (including SPK and PAK) has declined globally in recent years^5^. This may be due to the fact that the early transplant failure rate of pancreas transplantation is the highest among all solid organ transplants^6^. This is related to the surgical complexity of pancreas transplantation and susceptibility to ischemia-reperfusion injury^6^. Therefore, it is important to identify the risk factors for poor prognosis, which may guide the improvement of prognosis.

Nowadays, the vast majority of pancreas transplants are combined kidney transplants (including SPK and PAK), the proportion of pancreas transplantation alone is very small^6^. In addition, due to a shortage of donors, most patients must undergo renal replacement therapy with dialysis before receiving a transplant. Hemodialysis (HD) is the most commonly used form of dialysis worldwide, with ~89% of patients with end-stage kidney disease receiving HD^7^. However, peritoneal dialysis (PD) has advantages such as preserving residual kidney function, better quality of life, and lower cost compared to HD^8–10^. The choice of PD or HD is influenced by factors like patient autonomy, comorbidities, vascular or peritoneal diseases, and dialysis center factors.

PD appears to be a more effective transitional treatment for kidney transplant recipients prior to kidney transplantation. A recent systematic review revealed that kidney transplant recipients who underwent PD prior to transplantation had decreased risk of overall kidney graft failure and kidney delayed graft function^11^. While this study also encompassed some studies of SPK recipients, its primary focus was on kidney graft outcomes. Further investigation is needed to assess the outcomes of pancreas graft and specific complications associated with pancreas transplantation. Additionally, the unique nature of SPK or PAK in comparison to kidney transplantation alone (KTA) may also contribute to differences in the impact of dialysis modalities^1^.

The influence of pretransplant dialysis modalities (HD or PD) on prognosis in pancreas-kidney transplant recipients has been concerned and investigated. However, these studies have not reached a consistent conclusion. A previous study reported that compared with PD, pretransplantation HD is associated with decreased risk of pancreas graft failure, anastomotic leak, and thrombosis^12^. However, other studies have found that these outcomes are similar in patients undergoing pretransplant HD and those on PD^13,14^. These conflicting findings may be attributed to differences in selection criteria, sample size, or study design.

Therefore, the aim of this study was to conduct a systematic review and meta-analysis to determine the effects of pretransplant dialysis modalities on patients receiving SPK or PAK.

## Materials and methods

This systematic review was conducted according to the Preferred Reporting Items for Systematic Reviews and Meta-analyses 2020 (PRISMA 2020, Supplemental Digital Content 1, http://links.lww.com/JS9/C450, Supplemental Digital Content 2, http://links.lww.com/JS9/C451) reporting guideline and the Meta-analysis of Observational Studies in Epidemiology (MOOSE) reporting guideline^15,16^. The quality assessment was performed according to Assessing the Methodological Quality of Systematic Reviews (AMSTAR, Supplemental Digital Content 3, http://links.lww.com/JS9/C452)^17^. The protocol was prospectively registered in PROSPERO.

### Search strategy and selection of studies

Two researchers independently searched various studies and identified relevant full text from the following databases: PubMed, EMBASE, and Cochrane Library (from inception until 1 December 2023). Detailed retrieval is presented in Supplementary Materials (Table S1, Supplemental Digital Content 4 [SDC], http://links.lww.com/JS9/C453).

Detailed inclusion and exclusion criteria are present in Table S2, SDC (Supplemental Digital Content 4, http://links.lww.com/JS9/C453). In short, all studies investigating the relationship between dialysis modalities (HD and PD) and outcomes in pancreas-kidney transplant recipients (including SPK and PAK) were considered for inclusion, without limiting language. Clinical trials, cohort studies, and case–control studies are considered. In addition, in order to include as much relevant research as possible, conference abstracts, and letters with detailed data were also considered. Case reports, case series, cross-sectional studies, reviews, or studies without control groups were excluded. For studies with overlapping cohorts, consider the study with the most detailed data.

### Data extraction, methodological assessment, and outcome measures

Two researchers independently extracted data from the final included studies, and the differences were resolved by the third researchers. The extracted data included study characteristics, recipient characteristics, donor and transplant characteristics, and outcome data. The quality of the included studies was independently assessed by two researchers using the Newcastle–Ottawa scale (NOS)^18^. NOS score ≥8 points is considered as high quality.

The primary outcomes of the study were patient survival, pancreas graft survival, kidney graft survival, and postoperative intra-abdominal infection. Secondary outcomes included relaparotomy, pancreas graftectomy, graft thrombosis, bleeding complications, graft pancreatitis, anastomotic leak, rejection, kidney delayed graft function, cytomegalovirus infection, wound infection, and the length of hospital stay.

### Statistical analysis

Pooled hazard ratios (HRs) and 95% CIs were used to assess the effect of pretransplant dialysis modalities on the risk of mortality or pancreas/kidney graft loss. Use the adjusted HR if it is reported. For studies that did not report HR and 95% CI, we used the method described by Liu *et al*.^19^ to obtain survival data from the reported Kaplan–Meier curve and calculate it. Pooled intra-abdominal infections and other secondary outcomes, expressed as odds ratios (OR) and 95% CI, were calculated by including the number of total and events. Mean difference and 95% CI were used to pool length of hospital stay. Heterogeneity across studies was assessed by *I*² statistics, with significant heterogeneity defined as *I*²>50%. If the heterogeneity is significant, the random effects model is used. Otherwise, the common effect model is used.

Preplanned subgroup analyses were performed according to year of publication, study location, or sample size. Univariate meta-regression analysis was performed to explore the association between study characteristics, recipient characteristics, donor and transplant characteristics, and pooled primary outcomes. Sensitivity analysis is performed by iteratively omitting single studies from pooled analyses to assess their role in the pooled results of primary outcomes. Publication bias was assessed by Egger’ test and Begg’ test, with *P*<0.10^20,21^. All outcomes were analyzed using R software (version 4.2.1). *P-*values of less than 0.05 were considered statistically significant.

## Results

### Study selection

**Figure 1:**
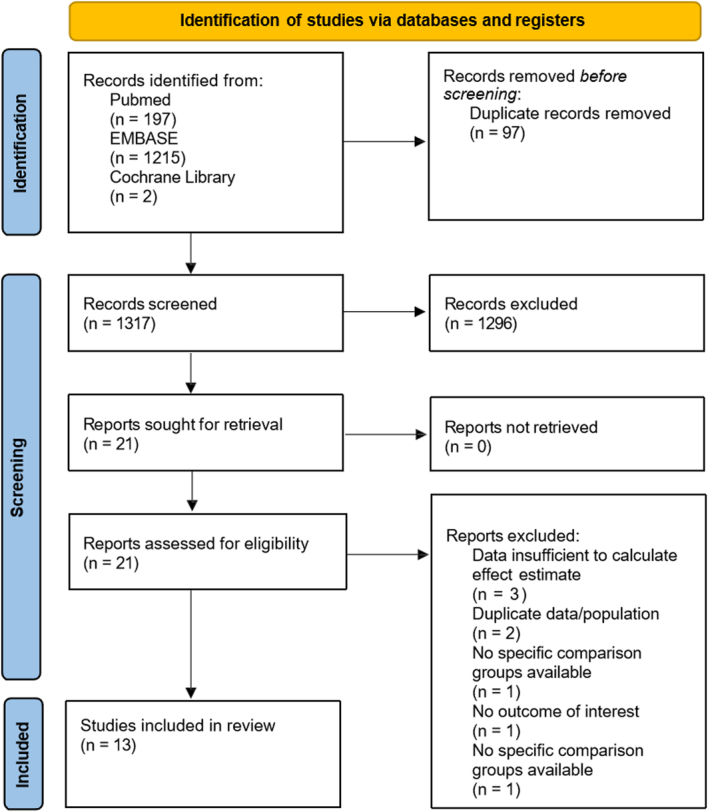
PRISMA flowchart.

A total of 1414 records were retrieved from the three databases, of which 97 were duplicates. After filtering the titles and abstracts of the remaining records, 1296 records were excluded. After eligibility determination of the full text of 21 studies, 8 studies were excluded. Finally, 13 studies were included in the systematic review and meta-analysis^12–14,22–31^. The PRISMA flowchart of the study selection is presented in Figure 1.

### Study characteristics and risk of bias

Table 1 and Table S3, SDC (Supplemental Digital Content 4, http://links.lww.com/JS9/C453) summarize the characteristics of the 13 included studies. The study sample size ranged from 27 to 200 and involved a total of 1503 pancreas-kidney transplant recipients. All studies were designed retrospectively. Most of the studies were conducted in SPK, and only one was in a mixed SPK and PAK cohort^12^. The mean recipient age ranged from 24.96 to 43.66 years, and the male proportion ranged from 48.1 to 72%. Dialysis duration ranged from 13 to 29.7 months, and diabetes duration ranged from 23.6 to 32.61 years. The NOS score ranges from 6 to 9, with five studies (38.46%) rated as high quality (Table S4, SDC, Supplemental Digital Content 4, http://links.lww.com/JS9/C453).

**Table 1:** Baseline characteristics of the included studies.

				Recipient characteristics					Donor characteristics		Transplant characteristics				
Study	Study site (study design)	Sample size (HD:PD)	Study period	Age	Male sex	BMI	Dialysis vintage (month)	Diabetes duration (year)	Age	Male sex	SPK or PAK	Exocrine drainage	Venous drainage	Cold ischemia time (hour)	Outcomes
Coffma (2023)^13^	USA (Retrospective cohort)	200 (130:70)	Nov 2001 - Aug 2020	24.96 ± 9.88	115 (57.5%)	24.3 ± 3.09	24.50 ± 21.58	25.13 ± 9.75	41.92 ± 9.26	139 (69.5%)	SPK	Enteric	Systemic 30 (15%); Portal 170 (85%)	Pancreas 16.08 ± 4.24；Kidney 15.15 ± 4.02	Patient survival, pancreas graft survival, kidney graft survival, intra-abdominal infection, relaparotomy, graft thrombosis, kidney delayed graft function, length of hospital stay
Surowiecka (2020)^30^	Poland (Retrospective cohort)	44 (33:11)	2006 - 2020	36	26 (60%)	NA	27.98 ± NA	NA	NA	NA	SPK	Enteric	Systemic	NA	Pancreas graftectomy
Scheuerman (2020)^14^	Germany (Retrospective cohort)	83 (64:19)	2000 - 2016	43.66 ± 9.18	46 (55.42%)	25.39 ± 4.28	29.70 ± 21.40	26.87 ± 8.40	22.98 ± 11.16	51 (61.45%)	SPK	Enteric	NA	Pancreas 11.02 ± 3.14； Kidney 10.28 ± 2.50	Patient survival, pancreas graft survival, kidney graft survival, intra-abdominal infection, relaparotomy, graft thrombosis, bleeding, graft pancreatitis, anastomotic leak, rejection, kidney delayed graft function, CMV infection, wound infection
Martinez (2020)^25^	Spain (Retrospective cohort)	27 (17:10)	2000 - 2019	NA	NA	NA	NA	NA	NA	NA	SPK	NA	NA	NA	Intra-abdominal infection, relaparotomy, graft thrombosis, rejection, kidney delayed graft function
Räihä (2019)^29^	Finland (Retrospective cohort)	96 (37:59)	Mar 2010 - Dec 2017	42.61 ± 8.37	NA	24.39 ± 2.47	13 ± 8.35	32.61 ± 8.37	38.77 ± 12.97	NA	SPK	Enteric	NA	Pancreas 10.14 ± 1.89；Kidney 8.16 ± 1.85	Pancreas graft survival, intra-abdominal infection, relaparotomy, bleeding, graft pancreatitis, rejection, kidney delayed graft function, length of hospital stay
Marcacuzco (2018)^24^	Spain (Retrospective cohort)	165 (98:67)	Mar 1995 - Dec 2015	38.9 ± 7.5	99 (60%)	23.7 ± 3.7	21 (13–29)	23.6 ± 7.5	29 (21-35)	111 (67%)	SPK	Enteric 106 (64%); Bladder 59 (36%)	Systemic	8.65 ± 1.9	Patient survival, pancreas graft survival, kidney graft survival, intra-abdominal infection, relaparotomy, pancreas graftectomy, graft thrombosis, bleeding, graft pancreatitis, anastomotic leak, rejection, CMV infection, wound infection, length of hospital stay
Martins (2015)^26^	Portugal (Retrospective cohort)	158 (119:39)	May 2000 - Dec 2013	34.88 ± 5.98	76 (48.10%)	22.27 ± 2.78	29.21 ± 21.04	23.87 ± 5.83	28.23 ± 10.44	NA	SPK	Enteric	Systemic	11.43 ± 4.93	Patient survival, relaparotomy, rejection, kidney delayed graft function, length of hospital stay
Ghazanfar (2012)^12^	UK (Retrospective cohort)	184 (88:96)	2001 - 2011	NA	NA	NA	NA	NA	NA	NA	SPK 160 (86.96%); PAK 24 (13.04%)	NA	NA	NA	Intra-abdominal infection, pancreas graftectomy, graft thrombosis, bleeding, anastomotic leak, rejection, wound infection
Ziaja (2011)^31^	Poland (Case–control)	46 (8:38)	2004 - 2010	37.3 ± 6.5	NA	22.6 ± 2.6	24.8 ± 18.8	24.5 ± 6.9	24.8 ± 6.9	NA	SPK	Enteric	Systemic	Pancreas 9.0 ± 2.0；Kidney 7.5 ± 1.8	Intra-abdominal infection
Padillo-Ruiz (2010)^27^	Spain (Retrospective cohort)	100 (75:25)	Jun 1996 - Feb 2009	39 ± 7.34	72 (72%)	NA	23.195 ± 14.49	24.05 ± 6.73	23.97 ± 8.96	NA	SPK	Enteric 82 (82%); Bladder 18 (18%)	Systemic	13.00 ± 5.65	Patient survival, pancreas graft survival, kidney graft survival, intra-abdominal infection
Kim (2005)^22^	Canada (Retrospective cohort)	120 (68:52)	Nov 1995 - Aug 2003	39 ± 6.06	72 (60%)	NA	21.7 ± 16.04	26 ± 6.54	NA	NA	SPK	Enteric 100 (83.33%); Bladder 20 (16.67%)	Systemic 76 (63.33%); Portal 44 (36.67%)	NA	Patient survival, pancreas graft survival, intra-abdominal infection
Malaise (2002)^23^	Belgium (Retrospective cohort)	177 (136:41)	May 1998 - Sep 2000	NA	NA	NA	NA	NA	NA	NA	SPK	NA	NA	NA	Intra-abdominal infection
Papalois (1996)^28^	USA (Retrospective cohort)	103 (71:32)	Jan 1986 - Dec 1994	38.84 ± 7.25	63 (61.17%)	NA	NA	25.06 ± 6.61	36.20 ± 15.41	NA	SPK	Bladder	Systemic	17.97 ± 4.92	Patient survival, pancreas graft survival, intra-abdominal infection

CMV, cytomegalovirus; HD, hemodialysis; NA, not applicable; PAK, pancreas after kidney transplantation; PD, peritoneal dialysis; SPK, simultaneous pancreas and kidney transplantation.

### Primary outcomes

**Figure 2:**
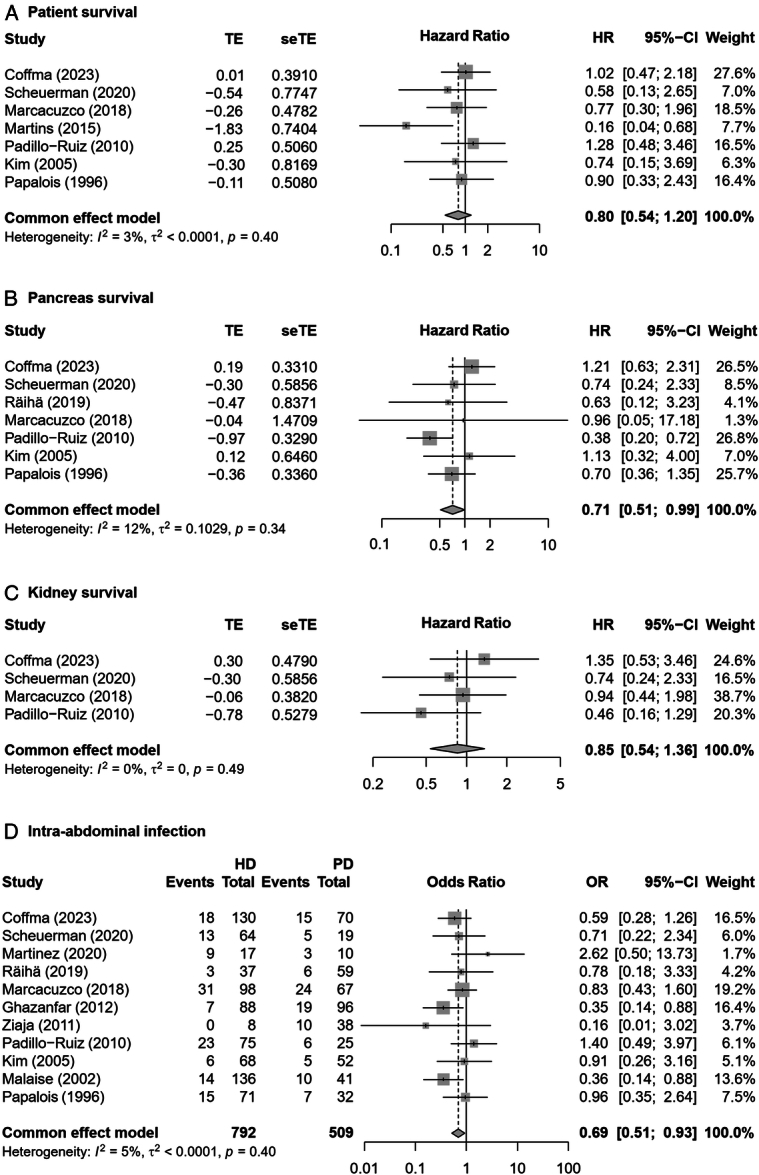
Forest plots on patient survival (A), pancreas survival (B), kidney survival (C), and intra-abdominal infection (D) in patients undergoing hemodialysis compared to peritoneal dialysis.

Compared with pretransplant PD, there was no significant difference in patient survival [HR, 0.80 (95% CI: 0.54–1.20); *I* ^2^=3%] (Fig. 2A)^13,14,22,24,26–28^ and kidney graft survival [HR, 0.85 (95% CI: 0.54–1.36); *I* ^2^=0%] (Fig. 2C)^13,14,24,27^ among HD patients. However, the pooled analysis of seven studies, involving 867 pancreas-kidney transplant recipients, revealed that pancreas graft survival was higher in patients who underwent HD compared to those on pretransplant PD [HR, 0.71 (95% CI: 0.51–0.99); *I* ^2^=12%] (Fig. 2B)^13,14,22,24,27–29^. Furthermore, pooled results from 1301 recipients demonstrated that pretransplant HD was associated with a decreased incidence of intra-abdominal infection [OR, 0.69 (95% CI: 0.51–0.93); *I* ^2^=5%] (Fig. 2D)^12–14,22–25,27–29,31^.

### Secondary outcomes

**Figure 3:**
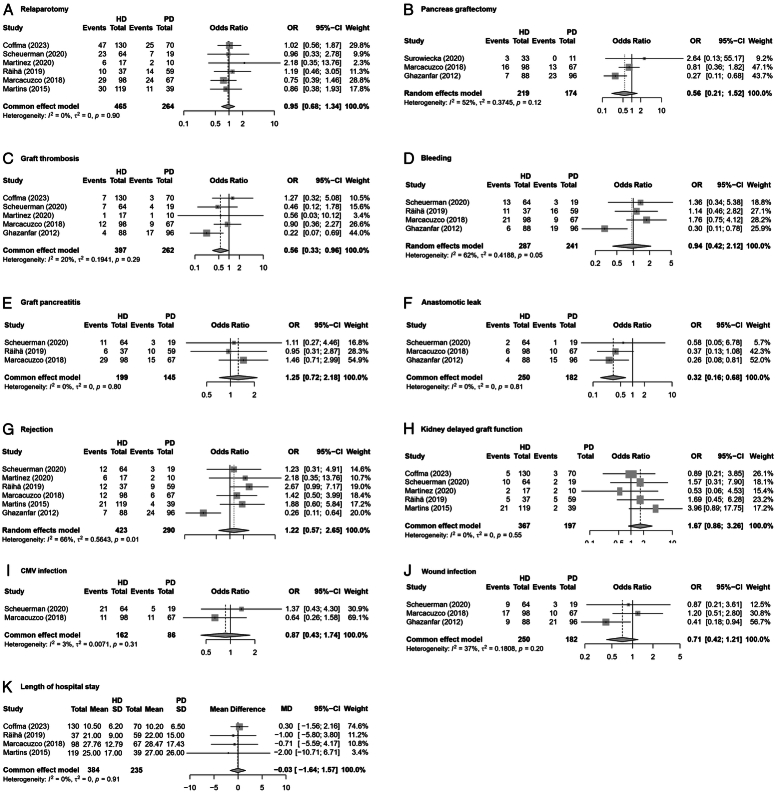
Forest plots on relaparotomy (A), pancreas graftectomy (B), graft thrombosis (C), bleeding (D), graft pancreatitis (E), anastomotic leak (F), rejection (G), kidney delayed graft function (H), cytomegalovirus infection (I), wound infection (J), and length of hospital stay (K) in patients undergoing hemodialysis compared to peritoneal dialysis.

A summary of pretransplant dialysis modality and secondary outcomes is presented in Figure 3. The pooled effect size showed no significant differences in relaparotomy, pancreas graftectomy, bleeding, graft pancreatitis, rejection, kidney delayed graft function, cytomegalovirus infection, wound infection, or length of hospital stay between patients who underwent PD prior to pancreas-kidney transplantation and those who underwent HD (Fig. 3A, B, D, E, G–K). Interestingly, pretransplant HD was associated with a decreased risk of graft thrombosis [OR, 0.56 (95% CI: 0.33–0.96); *I* ^2^=20%] (Fig. 3C)^12–14,24,25^ and anastomotic leak [OR, 0.32 (95% CI: 0.16–0.68); *I* ^2^=0%] (Fig. 3F)^12,14,24^ compared with PD.

### Subgroup analysis, meta-regression, sensitivity analysis, and publication bias

The association between pretransplant HD and pancreas graft survived was observed when subgroup analyses were based on studies published before 2015, studies with a sample size ≤100, or studies conducted in European. While the association between HD and intra-abdominal infection was observed when subgroup analyses were limited to studies published before 2015, studies with a sample size >100, or studies conducted in European. With respect to patient survival and kidney graft survival, no differences were observed between subgroups based on publication year, sample size, or study location (Table S5, SDC, Supplemental Digital Content 4, http://links.lww.com/JS9/C453).

According to univariate meta-regression analysis, the sample size of the study affected the risk of pancreas graft failure [estimate, −0.7171; (95% CI: −1.4006 to −0.0336); *P*=0.0398]. European studies have been associated with a lower risk of pancreas graft failure [estimate, −0.6925; (95% CI: −1.3722 to −0.0127); *P*=0.0459]. The proportion of male recipients was associated with a lower risk of pancreas graft failure [estimate, −0.0671; (95% CI: −0.1228 to −0.0115); *P*=0.0180], while donor age was associated with a higher risk of pancreas graft failure [estimate, 0.0483; (95% CI: 0.0035–0.0931); *P*=0.0180]. No significant covariates were found to affect the effects of dialysis modality on patient survival, kidney graft survival, and intra-abdominal infection. Meta-regression analysis of the primary outcomes was presented in Table S6, SDC (Supplemental Digital Content 4, http://links.lww.com/JS9/C453).

Sensitivity analyses for primary outcomes, patient survival and kidney graft survival were robust. Results for intra-abdominal infections are relatively robust and not affected by most studies. The outcome of pancreas graft survival was not robust (Table S7, SDC, Supplemental Digital Content 4, http://links.lww.com/JS9/C453).

The results of publication bias are presented in Table S8, SDC (Supplemental Digital Content 4, http://links.lww.com/JS9/C453). There may be publication bias in the analysis of length of hospital stay (*P*=0.0108 for Egger’ test, *P*=0.1742 for Begg’ test). In addition, no publication bias was found for other outcomes (both Begg’ and Egger’ tests >0.10).

## Discussion

This is the first systematic review and meta-analysis to summarize the effects of pretransplant dialysis modalities on pancreas-kidney transplant outcomes. The study found that patients who underwent HD prior to transplantation had better pancreas graft survival and decreased risk of intra-abdominal infection, anastomotic leak, and graft thrombosis compared with patients who underwent PD. However, the overall risk of bias in the included studies was moderate, and these results should be interpreted with caution.

The elevated risk of complications in PD patients may contribute to the decreased survival rate of pancreas grafts. Graft thrombosis is the primary cause of early pancreas graft loss, accounting for ~60% of graft losses within three months after transplantation^32^. Our study also revealed that patients who underwent PD prior to transplantation had a higher risk of graft thrombosis compared to patients who underwent HD. Graft thrombosis often leads to subsequent graft pancreatectomy^33^. Despite the findings of this study, no effect of dialysis modalities on kidney graft outcomes, including kidney delayed graft function and kidney graft survival, was found in pancreas-kidney transplant patients. However, this conclusion is based on limited studies and sample sizes. It is important to carefully consider the impact of dialysis modalities on kidney graft outcomes and conduct prospective studies with larger sample sizes, especially considering previous evidence that pretransplantation PD generally provides better preservation of residual native kidney function compared to HD^34–36^. Although this study did not find a significant difference in patient mortality among pancreas-kidney transplant recipients with different pretransplant dialysis modalities. However, previous studies have found that HD may increase the risk of cardiovascular events after renal transplantation through intermittent and nonphysiological volume changes^37^.

In contrast to KTA, SPK, or PAK procedures require abdominal manipulation, easier access to a stronger immunosuppressive regimen, and history of diabetes mellitus^1^. As a result, intra-abdominal infection is a common complication of these procedures^38^. The peritoneal catheter communicates with the skin and external environment, and long-term PD can lead to peritoneal fibrosis and decreased peritoneal defense function, potentially increasing the risk of intra-abdominal infection in PD patients^22,39^. Therefore, more frequent monitoring for infection in PD patients after surgery is recommend. Effective infection prevention programs need to be considered, but multidrug-resistant infections need to be vigilant. Furthermore, peritoneal thickening and fibrosis caused by long-term PD may lead to adhesions that affect anastomotic healing^39^. PD patients have an increased incidence of intraperitoneal infection and a secondary rupture of the anastomosis, potentially contributing to the increased incidence of anastomotic leak in PD patients.

Compared with kidney grafts, pancreas grafts are more susceptible to graft thrombosis^1,38^. In PD patients, the procoagulant system and blood concentration are thought to be more prone to thrombosis than in HD patients, potentially explaining the higher risk of graft thrombosis in PD patients^40,41^. However, potential confounding bias should be carefully considered. Patients with a hypercoagulable state or poor vascular condition before dialysis may be more likely to choose PD, making these patients themselves more susceptible to thrombosis. Our results suggest that active anticoagulant prophylaxis may be needed in patients with PD. However, this must be weighed against the increased risk of bleeding, although no difference in baseline bleeding risk was found between HD and PD patients in our study.

The current evidence is derived from retrospective studies and is of limited quality. However, it is currently challenging to conduct randomized clinical trials to compare the effects of HD and PD on the outcome of pancreas-kidney transplantation. In patients with diabetes and end-stage renal disease awaiting pancreas-kidney transplantation, we believe that HD is a reasonable replace treatment option. However, a comprehensive assessment of blood vessel and thrombosis status, as well as the patient’s willingness, needs to be considered. Therefore, nephrologists, transplant surgeons, endocrinologists, and patients need to make an integrated decision before initiating dialysis. For patients who received PD before transplantation, more potent infection prevention protocols and anticoagulant therapy regiments need to be considered to reduce the risk of intra-abdominal infection and graft thrombosis. Well-designed prospective studies are needed in future to validate the findings of this study.

This systematic review and meta-analysis must acknowledge certain limitations. Firstly, the included studies were designed retrospectively, and their limitations may introduce potential bias. Some studies excluded patients who had undergone dialysis switching, making it impossible to determine outcomes in this populations. Secondly, it is important to note the clinical heterogeneity between studies. The majority of the studies focused on SPK, with only one study including a mixed cohort of SPK and PAK. The studies also differed significantly in technical features, including endocrine and exocrine drainage. Therefore, caution should be exercised when interpreting the conclusions of this study in relation to other populations. However, despite significant clinical heterogeneity, statistical heterogeneity was not significant in most analyses, leading us to speculate that it has little effect on the outcome of dialysis mode. Thirdly, the data for intra-abdominal infection outcome and all secondary outcomes were not adjusted, potentially overlooking the influence of potential confounding factors and exaggerating the effect of dialysis modalities on outcomes. Lastly, the overall quality of the studies is moderate and uneven. Therefore, the results of this meta-analysis should be interpreted with caution.

## Conclusion

Compared with PD, patients who received HD before transplantation had comparable patient survival and kidney graft survival. However, patients who received HD had better pancreas graft survival and a decreased risk of abdominal infection, anastomotic leak, and graft thrombosis. The results of this study suggest that the outcome of pancreas-kidney transplantation in HD is better than that in PD. Unlike patients waiting for a kidney transplant alone, current evidence recommends HD as the preferred dialysis modality for patients waiting for pancreas-kidney transplantation. However, due to the limited quality of the evidence, it is necessary to conduct future prospective large sample studies.

## Ethical approval

This is a systematic review and meta-analysis and does not require ethical approval.

## Consent

No informed consent was involved.

## Sources of funding

This work was supported by the Natural Science Foundation of China (Grant number 81870513), the Key Research Funding for Sichuan Province (Grant number 2021YFS0118), and the Sichuan Science and Technology Program (Grant number 2023NSFSC0599).

## Author contribution

Y.L., T.L., and T.S.: conceived and designed the analysis and supervision and editing; Y.L. and Y.T.: collected the data; Y.L., Y.T., and Y.F.: contributed data or analysis tools; Y.L.: performed the analysis; Y.L.: wrote the paper.

## Conflicts of interest disclosure

The authors declare that they have no financial conflict of interest with regard to the content of this report.

## Research registration unique identifying number (UIN)

This systematic review protocol has been registered with PROSPERO under the registration number CRD42023493953. Link to https://www.crd.york.ac.uk/PROSPERO/display_record.php?RecordID=493953.

## Guarantor

Turun Song, MD, PhD and Yu Fan, MD.

## Data availability statement

We declared that materials described in the manuscript, including all relevant raw data, will be freely available to any scientist wishing to use them for non-commercial purposes, without breaching participant confidentiality.

## Provenance and peer review

Not commissioned, externally peer-reviewed.

## Supplementary Material

**Figure:**