# Supplementary material for: Effect of pretransplant dialysis modalities on pancreas-kidney transplant outcomes: a systematic review and meta-analysis
Source: Int J Surg. 2024 May 3;110(8):5078–86. doi: 10.1097/JS9.0000000000001542 (PMC11325998; doi:10.1097/JS9.0000000000001542)
Supplement: Supplementary file 3 [file js9-110-5078-s004.docx]

**Effect of pretransplant dialysis modalities on pancreas-kidney transplant outcomes: a systematic review and meta-analysis**

**Supplemental Digital Content**

**Table of contents**

Table S1. Search strategy1

Table S2. Study inclusion/exclusion criteria4

Table S3. Characteristics of included studies5

Table S4. Risk of bias assessment of included studies8

Table S5. Subgroup analysis of primary outcomes9

Table S6. Meta-regression of primary outcomes11

Table S7. Sensitivity analysis14

Table S8. Publication bias15

References16

**Table S1. Search strategy**

| **PubMed (From Inception to December 01, 2023)** | | |
| --- | --- | --- |
| **Search number** | Query | **Results** |
| #1 | (renal dialysis[MeSH Terms]) OR (dialysis[MeSH Terms]) OR (peritoneal dialysis, continuous ambulatory[MeSH Terms]) OR (dialysis, peritoneal[MeSH Terms]) OR (hemodialysis[MeSH Terms]) | 105,262 |
| #2 | (intradialy*[Title/Abstract]) OR (hemodialy*[Title/Abstract]) OR (hemofilt*[Title/Abstract]) OR (in-center dialysis[Title/Abstract]) OR (nocturnal dialysis[Title/Abstract]) OR (home dialysis[Title/Abstract]) OR (peritoneal dialysis[Title/Abstract]) OR (automated peritoneal dialysis[Title/Abstract]) OR (continuous ambulatory peritoneal dialysis[Title/Abstract]) OR (CAPD[Title/Abstract]) | 102,736 |
| #3 | #1 OR #2 | 178,271 |
| #4 | (renal transplantation[MeSH Terms]) OR (kidney transplantation[MeSH Terms]) OR (kidney transplant*[Title/Abstract]) OR (renal transplant*[Title/Abstract]) OR (kidney recipient[Title/Abstract]) OR (renal recipient[Title/Abstract]) | 126,766 |
| #5 | (pancreas Transplantation[MeSH Terms]) OR (pancreas transplant*[Title/Abstract]) OR (pancreas recipient[Title/Abstract]) | 8,813 |
| #6 | #3 AND #4 AND #5 | 197 |
| **EMBASE via OVID (From Inception to December 01, 2023)** | | |
| **Search number** | Query | **Results** |
| #1 | exp Renal Dialysis/ | 142,237 |
| #2 | ((end stage or endstage) adj (kidney or renal or dialysis)).tw,kw,rn. | 79,158 |
| #3 | ((kidney or renal) adj dialy*).tw,kw,rn. | 2,325 |
| #4 | (dialy* adj (patient* or therapy or modalit*)).tw,kw,rn. | 37,726 |
| #5 | exp H$emodialysis/ | 142,237 |
| #6 | (h$emodialy* or h$emofilt* or intradialy*).tw,kw,rn. | 120,288 |
| #7 | (in-center or incenter or nocturnal or home) adj3 (h$emodialy* or dialy*).tw,kw,rn. | 4,782 |
| #8 | exp Peritoneal Dialysis/ | 50,371 |
| #9 | *dialysis, peritoneal/ | 21,011 |
| #10 | (tenckhoff* automated or continuous or ambulatory) adj3 (peritoneal or dialy*).tw,kw,rn. | 10,108 |
| #11 | (peritoneal dialysis or pd or capd or ccpd or apd or ipd or nipd or tpd).tw,kw,rn. | 370,025 |
| #12 | or/1-11 | 601,131 |
| #13 | exp Kidney Transplantation/ | 184,374 |
| #14 | (kidney or renal) adj (transplant* or candidate* or organ or nephrop* or wait list* or recipient*).tw,kw,rn. | 146,630 |
| #15 | or/13-14 | 198,910 |
| #16 | exp Pancreas Transplantation/ | 22,639 |
| #17 | (Pancreas adj (transplant* or candidate* or organ or wait list* or recipient*)).tw,kw,rn. | 7,422 |
| #18 | or/16-17 | 23,340 |
| #19 | 12 and 15 and 18 | 1,215 |
| **Cochrane Library via OVID (From Inception to December 01, 2023)** | | |
| **Search number** | Query | **Results** |
| #1 | exp Renal Dialysis/ | 6,508 |
| #2 | ((end stage or endstage) adj (kidney or renal or dialysis)).tw,kw,rn. | 0 |
| #3 | ((kidney or renal) adj dialy*).tw,kw,rn. | 0 |
| #4 | (dialy* adj (patient* or therapy or modalit*)).tw,kw,rn. | 0 |
| #5 | exp H$emodialysis/ | 6,508 |
| #6 | (h$emodialy* or h$emofilt* or intradialy*).tw,kw,rn. | 0 |
| #7 | (in-center or incenter or nocturnal or home) adj3 (h$emodialy* or dialy*).tw,kw,rn. | 0 |
| #8 | exp Peritoneal Dialysis/ | 1,143 |
| #9 | *dialysis, peritoneal/ | 0 |
| #10 | (tenckhoff* automated or continuous or ambulatory) adj3 (peritoneal or dialy*).tw,kw,rn. | 0 |
| #11 | (peritoneal dialysis or pd or capd or ccpd or apd or ipd or nipd or tpd).tw,kw,rn. | 0 |
| #12 | or/1-11 | 6,508 |
| #13 | exp Kidney Transplantation/ | 4,439 |
| #14 | (kidney or renal) adj (transplant* or candidate* or organ or nephrop* or wait list* or recipient*).tw,kw,rn. | 0 |
| #15 | or/13-14 | 4,439 |
| #16 | exp Pancreas Transplantation/ | 161 |
| #17 | (Pancreas adj (transplant* or candidate* or organ or wait list* or recipient*)).tw,kw,rn. | 0 |
| #18 | or/16-17 | 161 |
| #19 | 12 and 15 and 18 | 2 |

**Table S2. Study inclusion/exclusion criteria**

| **Elements of PICOS** | **Criteria for Inclusion/Exclusion** |
| --- | --- |
| Populations | · SPK transplant recipients |
|  | · PAK transplant recipients |
| Interventions or Exposure | · Pretransplant transplant dialysis modalities: HD or PD |
| Control | · Another type of mode of pretransplant dialysis treatment |
| Outcomes | · Primary outcomes |
|  | v Patient survival |
|  | v Pancreas graft survival |
|  | v Kidney graft survival |
|  | v Intra-abdominal infection |
|  | · Secondary outcomes |
|  | v Other infection |
|  | v Relaparotomy |
|  | v Graft thrombosis (pancreas or kidney graft) |
|  | v Anastomotic leak (enteric or bladder) |
|  | v Rejection (pancreas or kidney graft) |
|  | v Kidney delayed graft function |
|  | v Pancreas graftectomy |
|  | v Graft pancreatitis |
|  | v Bleeding complication |
|  | v Length of hospital stay |
| Study design | · RCTs, cohort studies and case-control studies were included. |
|  | · Letters or conference abstracts with detailed data are also included. |
|  | · Cross-sectional, case series/case reports, review, and systematic review were exclued. |

Abbreviations: SPK, simultaneous pancreas and kidney transplantation; PAK, pancreas after kidney transplantation; HD, hemodialysis; PD, peritoneal dialysis; RCT, randomized controlled trial.

**Table S3. Characteristics of included studies**

| **Study** | **Institution** | **Pretransplant C-peptide** | **HbA1c pre-transplantation** | **Comorbidities** | **HLA Mismatch** | **PRA Titer** | **Donor CMV+/ recipient CMV−** | **Mean follow-up (month)** |
| --- | --- | --- | --- | --- | --- | --- | --- | --- |
| **Coffma (2023)^1^** | Atrium Health Wake Forest Baptist | ≥ 2.0 ng/mL: 47 | NA | NA | 4.54 ± 1.37 | PRA ≥ 20%: 12 | 50 | 112.5 ± 63.5 |
| **Surowiecka (2020)^2^** | Central Clinical Hospital of the Ministry of the Interior in Warsaw | NA | NA | NA | NA | NA | NA | NA |
| **Scheuerman (2020)^3^** | University Hospital of Leipzig | NA | 7.7 ± 1.67 | Diabetic retinopathy  69 (83.13%); Diabetic neuropathy  50 (60.24%); Arterial obstructive disease  14 (16.87%); Coronary heart disease  23 (27.71%); Depression  23 (27.71%) | NA | NA | CMV D +:  HD 31 (48.4%) , PD 11 (64.7%); CMV R +:  HD 36 (56.3%), PD 13 (68.4%) | NA |
| **Martinez (2020)^4^** | Hospital Clinico Universitario Virgen De La Arrixaca | NA | NA | NA | NA | NA | NA | NA |
| **Räihä (2019)^5^** | University of Helsinki and  Helsinki University Hospital | NA | NA | NA | HLA A/B 2.7 ± 0.99; HLA DR 1.44 ± 0.64 | NA | NA | 30.84 ± 21.20 |
| **Marcacuzco (2018)^6^** | Hospital Universitario Doce de Octubre | NA | 8 ± 1.3 | Retinopathy  157 (95.7%); Neuropathy  121 (73.8%); Cardiopathy  50 (30.5%); Gastroparesis  28 (17.1%); | NA | NA | 39 (23.6%) | NA |
| **Martins (2015)^7^** | Centro Hospitalar do Porto | NA | 9.05 ± 6.82;  HbA1c ≥ 9%: 50 | Cardiovascular disease 29 (18.35%) | 4.52 ± 1.08 | NA | NA | 70.44 ± 43.80 |
| **Ghazanfar (2012)^8^** | Central Manchester University Hospitals NHS Foundation Trust | NA | NA | NA | NA | NA | NA | NA |
| **Ziaja (2011)^9^** | Medical University of Silesia | NA | NA | NA | NA | NA | NA | NA |
| **Padillo-Ruiz (2010)^10^** | Reina Sofia Teaching Hospital | NA | NA | NA | NA | NA | NA | 55 ± 38 |
| **Kim (2005)^11^** | Toronto General Hospital, University of Toronto | NA | NA | NA | NA | NA | NA | 40.3 ± 26.93 |
| **Malaise (2002)^12^** | Universite´ Catholique de Louvain Ten European and one Israel transplant centers | NA | NA | NA | NA | NA | NA | NA |
| **Papalois (1996)^13^** | University of Minnesota, Minneapolis | NA | NA | NA | NA | NA | NA | NA |

Abbreviations: HbA1c, glycosylated hemoglobin A1c; HLA, human leukocyte antigen; PRA, panel reactive antibodies; CMV, cytomegalovirus; NA, not applicable; HD, hemodialysis; PD, peritoneal dialysis.

**Table S4. Risk of bias assessment of included studies**

| **Study** | **Selection** | | | | **Comparability** | **Outcomes** | | | **Total**  **NOS** |
| --- | --- | --- | --- | --- | --- | --- | --- | --- | --- |
|  | **Representativeness** | **Non-exposed: selection** | **Exposure: ascertainment** | **Outcomes:  not present at entry** | **Comparability of cohorts on the basis of the design or analysis** | **Assessment** | **Follow-up long enough** | **Adequacy of follow- up** |  |
| Coffma (2023)^1^ | 1 | 1 | 1 | 1 | 1 | 1 | 1 | 1 | 8 |
| Surowiecka (2020)^2^ | 1 | 1 | 0 | 1 | 1 | 1 | 1 | 1 | 7 |
| Scheuerman (2020)^3^ | 1 | 1 | 1 | 1 | 2 | 1 | 1 | 1 | 9 |
| Martinez (2020)^4^ | 1 | 1 | 0 | 1 | 0 | 1 | 1 | 1 | 6 |
| Räihä (2019)^5^ | 1 | 1 | 0 | 1 | 1 | 1 | 1 | 1 | 7 |
| Marcacuzco (2018)^6^ | 1 | 1 | 1 | 1 | 2 | 1 | 1 | 1 | 9 |
| Martins (2015)^7^ | 1 | 1 | 1 | 1 | 2 | 1 | 1 | 1 | 9 |
| Ghazanfar (2012)^8^ | 1 | 1 | 0 | 1 | 0 | 1 | 1 | 1 | 6 |
| Ziaja (2011)^9^ | 1 | 1 | 0 | 1 | 1 | 1 | 1 | 1 | 7 |
| Padillo-Ruiz (2010)^10^ | 1 | 1 | 0 | 1 | 1 | 1 | 1 | 1 | 7 |
| Kim (2005)^11^ | 1 | 1 | 1 | 1 | 1 | 1 | 1 | 1 | 8 |
| Malaise (2002)^12^ | 1 | 1 | 0 | 1 | 1 | 1 | 1 | 1 | 7 |
| Papalois (1996)^13^ | 1 | 1 | 0 | 1 | 1 | 1 | 1 | 1 | 7 |

**Table S5. Subgroup analysis of primary outcomes**

| **Subgroup comparison: Patient survival (HD vs. PD)** | **No. of Studies** | **No. of Participants** | **HR (95% CI)** | **Heterogeneity** | | | |
| --- | --- | --- | --- | --- | --- | --- | --- |
|  |  |  |  | ***Q* Statistic** | ***P* Value** | ***I^2^* Index (95% CI)** | ***τ^2^*** |
| **Publication Date** |  |  |  |  |  |  |  |
| Before 2015 | 3^10,11,13^ | 323 | 1.0120 (0.5318 - 1.9258) | 0.42 | 0.81 | 0.00% | 0.0000 |
| 2015 to 2023 | 4^1,3,6,7^ | 606 | 0.6926 (0.4132 - 1.1608) | 4.96 | 0.18 | 39.50% | 0.1859 |
| **Sample Size** |  |  |  |  |  |  |  |
| ≤100 | 2^3,10^ | 183 | 1.0131 (0.4417 - 2.3242) | 0.73 | 0.39 | 0.00% | 0.0000 |
| >100 | 5^1,6,7,11,13^ | 746 | 0.7482 (0.4721 - 1.1859) | 5.06 | 0.28 | 21.00% | <0.0001 |
| **Study Location** |  |  |  |  |  |  |  |
| European | 4^3,6,7,10^ | 506 | 0.6874 (0.3882 - 1.2171) | 5.49 | 0.14 | 45.30% | 0.3006 |
| Non-European region/international | 3^1,11,13^ | 423 | 0.9376 (0.5314 - 1.6543) | 0.13 | 0.94 | 0.00% | 0.0000 |
| **Subgroup comparison: Pancreas survival (HD vs. PD)** | **No. of Studies** | **No. of Participants** | **HR (95% CI)** | **Heterogeneity** | | | |
|  |  |  |  | ***Q* Statistic** | ***P* Value** | ***I^2^* Index (95% CI)** | ***τ^2^*** |
| **Publication Date** |  |  |  |  |  |  |  |
| Before 2015 | 3^10,11,13^ | 323 | 0.5596 (0.3630 - 0.8629) | 3.02 | 0.22 | 33.70% | 0.0822 |
| 2015 to 2023 | 4^1,3,5,6^ | 544 | 1.0126 (0.5989 - 1.7118) | 0.90 | 0.82 | 0.00% | 0.0000 |
| **Sample Size** |  |  |  |  |  |  |  |
| ≤100 | 3^3,5,10^ | 279 | 0.4608 (0.2707 - 0.7844) | 1.15 | 0.56 | 0.00% | 0.0000 |
| >100 | 4^1,6,11,13^ | 588 | 0.9440 (0.6145 - 1.4502) | 1.46 | 0.69 | 0.00% | 0.0000 |
| **Study Location** |  |  |  |  |  |  |  |
| European | 4^3,5,6,10^ | 444 | 0.4721 (0.2798 - 0.7966) | 1.39 | 0.71 | 0.00% | 0.0000 |
| Non-European region/international | 3^1,11,13^ | 423 | 0.9436 (0.6113 - 1.4566) | 1.46 | 0.48 | 0.00% | 0.0032 |
| **Subgroup comparison: Kidney survival (HD vs. PD)** | **No. of Studies** | **No. of Participants** | **HR (95% CI)** | **Heterogeneity** | | | |
|  |  |  |  | ***Q* Statistic** | ***P* Value** | ***I^2^* Index (95% CI)** | ***τ^2^*** |
| **Publication Date** |  |  |  |  |  |  |  |
| Before 2015 | 1^10^ | 100 | 0.4570 (0.1624 - 1.2862) | 0.00 | NA | NA | NA |
| 2015 to 2023 | 3^1,3,6^ | 448 | 0.9997 (0.5935 - 1.6840) | 0.69 | 0.71 | 0.00% | 0.0000 |
| **Sample Size** |  |  |  |  |  |  |  |
| ≤100 | 2^3,10^ | 183 | 0.5675 (0.2631 - 1.2238) | 0.38 | 0.54 | 0.00% | 0.0000 |
| >100 | 2^1,6^ | 365 | 1.0808 (0.6019 - 1.9407) | 0.36 | 0.55 | 0.00% | 0.0000 |
| **Study Location** |  |  |  |  |  |  |  |
| European | 3^3,6,10^ | 348 | 0.7340 (0.4293 - 1.2549) | 1.21 | 0.54 | 0.00% | 0.0000 |
| Non-European region/international | 1^1^ | 200 | 1.3526 (0.5290 - 3.4584) | 0.00 | NA | NA | NA |
| **Subgroup comparison: Intra-abdominal infection (HD vs. PD)** | **No. of Studies** | **No. of Participants** | **OR (95% CI)** | **Heterogeneity** | | | |
|  |  |  |  | ***Q* Statistic** | ***P* Value** | ***I^2^* Index (95% CI)** | ***τ^2^*** |
| **Publication Date** |  |  |  |  |  |  |  |
| Before 2015 | 6^8-13^ | 446 | 0.6024 (0.3930 - 0.9233) | 7.17 | 0.21 | 30.20% | 0.1558 |
| 2015 to 2023 | 5^1,3-6^ | 346 | 0.7928 (0.5209 - 1.2066) | 2.65 | 0.62 | 0.00% | 0.0000 |
| **Sample Size** |  |  |  |  |  |  |  |
| ≤100 | 5^3-5,9,10^ | 201 | 0.9805 (0.5424 - 1.7722) | 3.62 | 0.46 | 0.00% | <0.0001 |
| >100 | 6^1,6,8,11-13^ | 591 | 0.6132 (0.4332 - 0.8681) | 4.77 | 0.45 | 0.00% | 0.0000 |
| **Study Location** |  |  |  |  |  |  |  |
| European | 7^3-6,8-10^ | 387 | 0.7492 (0.5074 - 1.1062) | 7.36 | 0.29 | 18.40% | 0.0536 |
| Non-European region/international | 4^1,11-13^ | 405 | 0.6177 (0.3881 - 0.9831) | 2.53 | 0.47 | 0.00% | 0.0000 |

Abbreviations: HD, hemodialysis; PD, peritoneal dialysis; HR, hazard ratio; CI, confidence interval; OR, odds ratio.

**Table S6. Meta-regression of primary outcomes**

| **Covariate** | **Patient survival** | | | |
| --- | --- | --- | --- | --- |
|  | **No. of Studies** | **Estimate (95% CI)** | ***Z* Value** | ***P* Value** |
| **Study Characteristics** |  |  |  |  |
| Proportion of PD modality (per %) | 7^1,3,6,7,10,11,13^ | 0.0166 (-0.0430 to 0.0763) | 0.5465 | 0.5847 |
| Publication date (before 2015 vs. 2015 to 2023) | 7^1,3,6,7,10,11,13^ | 0.3792 (-0.4458 to 1.2043) | 0.9008 | 0.3677 |
| Sample size (≤100 vs. >100) | 7^1,3,6,7,10,11,13^ | 0.3031 (-0.6464 to 1.2526) | 0.6257 | 0.5315 |
| Study location (European vs. non-European region/international | 7^1,3,6,7,10,11,13^ | -0.3104 (-1.1159 to 0.4951) | -0.7553 | 0.4500 |
| **Recipient Characteristics** |  |  |  |  |
| Recipient age (mean, per 1 year) | 7^1,3,6,7,10,11,13^ | -0.012 (-0.0741 to 0.0502) | -0.3779 | 0.7055 |
| Male (per %) | 7^1,3,6,7,10,11,13^ | 0.0623 (-0.003 to 0.1276) | 1.8688 | 0.0616 |
| BMI, (mean, per 1 kg/m2) | 4^1,3,6,7^ | 0.4992 (-0.1314 to 1.1298) | 1.5515 | 0.1208 |
| Diabetes vintage (mean, per 1 year) | 7^1,3,6,7,10,11,13^ | 0.0468 (-0.3887 to 0.4824) | 0.2108 | 0.8330 |
| Dialysis vintage (mean, per 1 month) | 6^1,3,6,7,10,11^ | -0.1176 (-0.2751 to 0.040) | -1.4628 | 0.1435 |
| **Donor and Transplant Characteristics** |  |  |  |  |
| Donor age (mean, per 1 year) | 6^1,3,6,7,10,13^ | 0.0170 (-0.0474 to 0.0814) | 0.5174 | 0.6049 |
| Male (per %) | 3^1,3,6^ | 0.0722 (-0.1366 to 0.2809) | 0.6776 | 0.4980 |
| Pancreas cold ischemia time (mean, per 1 hour) | 6^1,3,6,7,10,13^ | 0.0547 (-0.0712 to 0.1806) | 0.8516 | 0.3945 |
| Kidney cold ischemia time (mean, per 1 hour) | 6^1,3,6,7,10,13^ | 0.055 (-0.0762 to 0.1861) | 0.8213 | 0.4115 |
| Enteric exocrine drainage | 7^1,3,6,7,10,11,13^ | -0.0025 (-0.0143 to 0.0094) | -0.4064 | 0.6844 |
| Systemic venous drainage | 6^1,6,7,10,11,13^ | -0.0036 (-0.016 to 0.0087) | -0.5771 | 0.5639 |
| **Covariate** | **Pancreas graft survival** | | | |
|  | **No. of Studies** | **Estimate (95% CI)** | ***Z* Value** | ***P* Value** |
| **Study Characteristics** |  |  |  |  |
| Proportion of PD modality (per %) | 7^1,3,5,6,10,11,13^ | 0.0206 (-0.0237 to 0.0649) | 0.9103 | 0.3627 |
| Publication date (before 2015 vs. 2015 to 2023) | 7^1,3,5,6,10,11,13^ | -0.5601 (-1.3016 to 0.1815) | -1.4803 | 0.1388 |
| Sample size (≤100 vs. >100) | 7^1,3,5,6,10,11,13^ | -0.7171 (-1.4006 to -0.0336) | -2.0563 | 0.0398 |
| Study location (European vs. non-European region/international | 7^1,3,5,6,10,11,13^ | -0.6925 (-1.3722 to -0.0127) | -1.9967 | 0.0459 |
| **Recipient Characteristics** |  |  |  |  |
| Recipient age (mean, per 1 year) | 7^1,3,5,6,10,11,13^ | -0.0424 (-0.0995 to 0.0147) | -1.4547 | 0.1458 |
| Male (per %) | 6^1,3,6,10,11,13^ | -0.0671 (-0.1228 to -0.0115) | -2.3653 | 0.0180 |
| BMI, (mean, per 1 kg/m2) | 4^1,3,5,6^ | -0.3489 (-1.4836 to 0.7858) | -0.6027 | 0.5467 |
| Diabetes vintage (mean, per 1 year) | 7^1,3,5,6,10,11,13^ | 0.0308 (-0.1900 to 0.2516) | 0.2738 | 0.7843 |
| Dialysis vintage (mean, per 1 month) | 6^1,3,5,6,10,11^ | 0.0131 (-0.1242 to 0.1505) | 0.1874 | 0.8514 |
| **Donor and Transplant Characteristics** |  |  |  |  |
| Donor age (mean, per 1 year) | 6^1,3,5,6,10,13^ | 0.0483 (0.0035 to 0.0931) | 2.1148 | 0.0340 |
| Male (per %) | 3^1,3,6^ | 0.0610 (-0.1027 to 0.2247) | 0.7305 | 0.4651 |
| Pancreas cold ischemia time (mean, per 1 hour) | 6^1,3,5,6,10,13^ | 0.0510 (-0.1187 to 0.2206) | 0.5888 | 0.5560 |
| Kidney cold ischemia time (mean, per 1 hour) | 6^1,3,5,6,10,13^ | 0.0288 (-0.1319 to 0.1894) | 0.3511 | 0.7255 |
| Enteric exocrine drainage | 7^1,3,5,6,10,11,13^ | 0.0018 (-0.0106 to 0.0141) | 0.2784 | 0.7807 |
| Systemic venous drainage | 5^1,6,10,11,13^ | -0.0101 (-0.0207 to 0.0004) | -1.8797 | 0.0602 |
| **Covariate** | **Kidney graft survival** | | | |
|  | **No. of Studies** | **Estimate (95% CI)** | ***Z* Value** | ***P* Value** |
| **Study Characteristics** |  |  |  |  |
| Proportion of PD modality (per %) | 4^1,3,6,10^ | 0.0329 (-0.0310 to 0.0968) | 1.0101 | 0.3124 |
| Publication date (before 2015 vs. 2015 to 2023) | 4^1,3,6,10^ | -0.7828 (-1.9415 to 0.3759) | -1.3241 | 0.1855 |
| Sample size (≤100 vs. >100) | 4^1,3,6,10^ | -0.6443 (-1.6103 to 0.3218) | -1.3071 | 0.1912 |
| Study location (European vs. non-European region/international | 4^1,3,6,10^ | -0.6112 (-1.6924 to 0.4699) | -1.1081 | 0.2678 |
| **Recipient Characteristics** |  |  |  |  |
| Recipient age (mean, per 1 year) | 4^1,3,6,10^ | -0.0382 (-0.1079 to 0.0315) | -1.0734 | 0.2831 |
| Male (per %) | 4^1,3,6,10^ | -0.0513 (-0.1323 to 0.0297) | -1.2407 | 0.2147 |
| BMI, (mean, per 1 kg/m2) | 3^1,3,6^ | -0.1171 (-0.9260 to 0.6919) | -0.2836 | 0.7767 |
| Diabetes vintage (mean, per 1 year) | 4^1,3,6,10^ | 0.0309 (-0.3924 to 0.4541) | 0.1429 | 0.8864 |
| Dialysis vintage (mean, per 1 month) | 4^1,3,6,10^ | -0.0089 (-0.1746 to 0.1568) | -0.1054 | 0.9160 |
| **Donor and Transplant Characteristics** |  |  |  |  |
| Donor age (mean, per 1 year) | 4^1,3,6,10^ | 0.0441 (-0.0212 to 0.1095) | 1.3234 | 0.1857 |
| Male (per %) | 3^1,3,6^ | 0.0708 (-0.1119 to 0.2535) | 0.7595 | 0.4476 |
| Pancreas cold ischemia time (mean, per 1 hour) | 4^1,3,6,10^ | 0.0295 (-0.1524 to 0.2114) | 0.3183 | 0.7502 |
| Kidney cold ischemia time (mean, per 1 hour) | 4^1,3,6,10^ | 0.0228 (-0.1791 to 0.2247) | 0.2216 | 0.8246 |
| Enteric exocrine drainage | 4^1,3,6,10^ | 0.0042 (-0.0295 to 0.0379) | 0.2424 | 0.8084 |
| Systemic venous drainage | 3^1,6,10^ | -0.0075 (-0.0220 to 0.007) | -1.0077 | 0.3136 |
| **Covariate** | **Intra-abdominal infection** | | | |
|  | **No. of Studies** | **Estimate (95% CI)** | ***Z* Value** | ***P* Value** |
| **Study Characteristics** |  |  |  |  |
| Proportion of PD modality (per %) | 11^1,3-6,8-13^ | -0.0103 (-0.0379 to 0.0172) | -0.7351 | 0.4623 |
| Publication date (before 2015 vs. 2015 to 2023) | 11^1,3-6,8-13^ | -0.2552 (-0.8922 to 0.3817) | -0.7854 | 0.4322 |
| Sample size (≤100 vs. >100) | 11^1,3-6,8-13^ | 0.5269 (-0.1873 to 1.2412) | 1.4459 | 0.1482 |
| Study location (European vs. non-European region/international | 11^1,3-6,8-13^ | 0.2398 (-0.3896 to 0.8691) | 0.7467 | 0.4553 |
| **Recipient Characteristics** |  |  |  |  |
| Recipient age (mean, per 1 year) | 8^1,3,5,6,9-11,13^ | 0.0237 (-0.0327 to 0.0801) | 0.8235 | 0.4102 |
| Male (per %) | 6^1,3,6,10,11,13^ | 0.0507 (-0.0291 to 0.1304) | 1.2442 | 0.2134 |
| BMI, (mean, per 1 kg/m2) | 5^1,3,5,6,9^ | 0.0383 (-0.6867 to 0.7633) | 0.1036 | 0.9175 |
| Diabetes vintage (mean, per 1 year) | 8^1,3,5,6,9-11,13^ | -0.0208 (-0.1882 to 0.1465) | -0.2441 | 0.8072 |
| Dialysis vintage (mean, per 1 month) | 7^1,3,5,6,9-11^ | -0.0190 (-0.1227 to 0.0846) | -0.3599 | 0.7190 |
| **Donor and Transplant Characteristics** |  |  |  |  |
| Donor age (mean, per 1 year) | 7^1,3,5,6,9,10,13^ | -0.0197 (-0.0729 to 0.0335) | -0.7248 | 0.4686 |
| Male (per %) | 3^1,3,6^ | -0.0256 (-0.2006 to 0.1494) | -0.2868 | 0.7742 |
| Pancreas cold ischemia time (mean, per 1 hour) | 7^1,3,5,6,9,10,13^ | -0.0023 (-0.1067 to 0.1021) | -0.0431 | 0.9656 |
| Kidney cold ischemia time (mean, per 1 hour) | 7^1,3,5,6,9,10,13^ | 0.007 (-0.0982 to 0.1122) | 0.1303 | 0.8963 |
| Enteric exocrine drainage | 8^1,3,5,6,9-11,13^ | -0.0039 (-0.0152 to 0.0075) | -0.6667 | 0.5050 |
| Systemic venous drainage | 6^1,6,9-11,13^ | 0.005- (-0.0055 to 0.0155) | 0.9418 | 0.3463 |

Abbreviations: PD, peritoneal dialysis; BMI, body mass index.

**Table S7. Sensitivity analysis**

| **Study** | **HR (95% CI)** | | | **OR (95% CI)** |
| --- | --- | --- | --- | --- |
|  | **Patient survival** | **Pancreas graft survival** | **Kidney graft survival** | **Intra-abdominal infection** |
| **All studies** | **0.8035 (0.5372 - 1.2020)** | **0.7114 (0.5094 - 0.9936)** | **0.8532 (0.5355 - 1.3591)** | **0.6931 (0.5139 - 0.9348)** |
| Coffma (2023)^1^ | 0.7350 (0.4578 - 1.1800) | 0.5875 (0.3979 - 0.8675) | 0.7340 (0.4293 - 1.2549) | 0.7137 (0.5154 - 0.9882) |
| Surowiecka (2020)^2^ | NA | NA | NA | NA |
| Scheuerman (2020)^3^ | 0.8235 (0.5423 - 1.2504) | 0.7088 (0.4999 - 1.0050) | 0.8772 (0.5271 - 1.4601) | 0.6918 (0.5079 - 0.9423) |
| Martinez (2020)^4^ | NA | NA | NA | 0.6588 (0.4855 - 0.8939) |
| Räihä (2019)^5^ | NA | 0.7153 (0.5085 - 1.0062) | NA | 0.6894 (0.5079 - 0.9357) |
| Marcacuzco (2018)^6^ | 0.8115 (0.5195 - 1.2677) | 0.7085 (0.5062 - 0.9918) | 0.8041 (0.4437 - 1.4574) | 0.6609 (0.4722 - 0.9250) |
| Martins (2015)^7^ | 0.9192 (0.6044 - 1.3978) | NA | NA | NA |
| Ghazanfar (2012)^8^ | NA | NA | NA | 0.7606 (0.5526 - 1.0469) |
| Ziaja (2011)^9^ | NA | NA | NA | 0.7134 (0.5273 - 0.9651) |
| Padillo-Ruiz (2010)^10^ | 0.7325 (0.4714 - 1.1382) | 0.8968 (0.6069 - 1.3253) | 0.9997 (0.5935 - 1.6840) | 0.6469 (0.4724 - 0.8857) |
| Kim (2005)^11^ | 0.8077 (0.5328 - 1.2246) | 0.6873 (0.4861 - 0.9717) | NA | 0.6815 (0.5008 - 0.9276) |
| Malaise (2002)^12^ | NA | NA | NA | 0.7460 (0.5435 - 1.0240) |
| Papalois (1996)^13^ | 0.7865 (0.5063 - 1.2216) | 0.7168 (0.4864 - 1.0561) | NA | 0.6718 (0.4909 - 0.9193) |

Abbreviations: HR, hazard ratio; CI, confidence interval; OR, odds ratio; NA, not applicable.**Table S8. Publication bias**

| **Outcomes** | **No. of Studies** | ***P* Value for Begg’s Test** | ***P* Value for Egger’s Test** |
| --- | --- | --- | --- |
| **Primary Outcomes** |  |  |  |
| Patient survival | 7^1,3,6,7,10,11,13^ | 0.2931 | 0.1327 |
| Pancreas graft survival | 7^1,3,5,6,10,11,13^ | 0.6523 | 0.7298 |
| Kidney graft survival | 4^1,3,6,10^ | 0.4969 | 0.5723 |
| Intra-abdominal infection | 11^1,3-6,8-13^ | 0.4835 | 0.7868 |
| **Secondary Outcomes** |  |  |  |
| Relaparotomy | 6^1,3-7^ | 0.1885 | 0.1288 |
| Pancreas graftectomy | 3^2,6,8^ | 0.6015 | 0.726 |
| Graft thrombosis | 5^1,3,4,6,8^ | 0.6242 | 0.9367 |
| Bleeding | 4^3,5,6,8^ | 0.4969 | 0.9262 |
| Graft pancreatitis | 3^3,5,6^ | 0.6015 | 0.3866 |
| Anastomotic leak | 3^3,6,8^ | 0.6015 | 0.5568 |
| Rejection | 6^3-8^ | 0.851 | 0.4125 |
| Kidney delayed graft function | 5^1,3-5,7^ | 0.3272 | 0.3973 |
| CMV infection | 2^3,6^ | NA | NA |
| Wound infection | 3^3,6,8^ | 0.6015 | 0.8827 |
| Length of hospital stay | 4^1,5-7^ | 0.1742 | 0.0108 |

Abbreviations: CMV, cytomegalovirus.

**References**

1. Coffman D, Jay CL, McCracken E, et al. Does dialysis modality or duration influence outcomes in simultaneous pancreas-kidney transplant recipients? Single center experience and review of the literature. *Clin Transplant.* 2023;37(6):e15009.

2. Surowiecka A, Matejak-Gorska M, Durlik M. Influence of peritoneal or hemodialysis on results of simultaneous pancreas and kidney transplant. *Experimental and Clinical Transplantation.* 2020;18(1):8-12.

3. Scheuermann U, Rademacher S, Jahn N, et al. Impact of pre-transplant dialysis modality on the outcome and health-related quality of life of patients after simultaneous pancreas-kidney transplantation. *Health Qual Life Outcomes.* 2020;18(1):303.

4. Martinez A, Lanuza M, Manzano D, et al. Impact of mode of dialysis on complications after simultaneous pancreas-kidney transplantation. *Nephrology Dialysis Transplantation.* 2020;35(SUPPL 3):iii1481.

5. Räihä J, Helanterä I, Ekstrand A, Nordin A, Sallinen V, Lempinen M. Effect of Pretransplant Dialysis Modality on Outcomes After Simultaneous Pancreas-Kidney Transplantation. *Ann Transplant.* 2019;24:426-431.

6. Marcacuzco A, Jiménez-Romero C, Manrique A, et al. Outcome of patients with hemodialysis or peritoneal dialysis undergoing simultaneous pancreas-kidney transplantation. Comparative study. *Clin Transplant.* 2018;32(6):e13268.

7. Martins LS, Malheiro J, Pedroso S, et al. Pancreas-Kidney transplantation: Impact of dialysis modality on the outcome. *Transpl Int.* 2015;28(8):972-979.

8. Ghazanfar A, Pretorian OM, Masood O, et al. The impact of the modality of dialysis on pancreas transplant outcomes. A single centre experience. *American Journal of Transplantation.* 2012;3):157-158.

9. Ziaja J, Krol R, Chudek J, et al. Intra-abdominal infections after simultaneous pancreas - kidney transplantation. *Ann Transplant.* 2011;16(3):36-43.

10. Padillo-Ruiz J, Arjona-Sánchez A, Muñoz-Casares C, Ruiz-Rabelo J, Navarro MD, Regueiro JC. Impact of peritoneal dialysis versus hemodialysis on incidence of intra-abdominal infection after simultaneous pancreas-kidney transplant. *World J Surg.* 2010;34(7):1684-1688.

11. Kim RD, Oreopoulos DG, Qiu K, et al. Impact of mode of dialysis on intra-abdominal infection after simultaneous pancreas-kidney transplantation. *Transplantation.* 2005;80(3):339-343.

12. Malaise J, Squifflet JP, Van Ophem D, Group ES. Influence of the type of renal replacement therapy on peritonitis rate after simultaneous pancreas kidney transplantation. *Transplantation proceedings.* 2002;Vol.34(7):2823p.

13. Papalois BE, Troppmann C, Gruessner AC, Benedetti E, Sutherland DER, Gruessner RWG. Long-term peritoneal dialysis before transplantation and intra-abdominal infection after simultaneous pancreas-kidney transplantations. *Archives of Surgery.* 1996;131(7):761-766.
